# An Indian pediatric emergency weight estimation tool: prospective adjustment of the Broselow tape

**DOI:** 10.1186/s12245-015-0078-z

**Published:** 2015-08-04

**Authors:** Farhad Asskaryar, Ravi Shankar

**Affiliations:** Department of Community Medicine, Sri Ramachandra University, College of Medicine & Research Institute, Porur, Chennai, 600116 Tamil Nadu India

**Keywords:** Broselow tape, Adjustment, Pediatric supine heights, Weights

## Abstract

**Background:**

This study aims to remodel the Broselow Pediatric Emergency Tape for the Indian pediatric population. The Broselow tape overestimates the heights of the Indian pediatric population and remits inaccurate predicted weights for all color zones with varying degrees and could result in overresuscitation of Indian children in emergency settings. The Indian children are underweight for their age and height.

**Methods:**

We prospectively collected cross-sectional data on a sample of 1185 children aged 1 month to 12 years old in Chennai, India. The Broselow tape was used for length-based weight estimation, and actual weight was recorded by a weighing scale. In the first stage, we recruited 769 children. With univariate linear regression, we adjusted the Broselow tape by an 8 % correction factor to enhance accuracy and created a new tape with new weight and height ranges. In the second stage, we recruited 416 children and tested the new ranges for accuracy.

**Results:**

The Broselow tape overestimates weights with a mean percentage difference of 5–15 % depending on the color zone. Accuracy of the Broselow tape by color-coded zone was between 33–86.6 %, with higher weight color zones showing lower accuracy. The new Indian pediatric weight estimation tool (IPWET), based on the Broselow tape has a weight range of 4–36 kg and height range of 50–150 cm (Broselow tape, 3–36 kg, 46–146.5 cm) and an improved accuracy between 51–97.8 %.

**Conclusions:**

A remodeled Broselow tape can predict weights with higher accuracy in the Indian pediatric population.

## Background

The need for the use of the Broselow tape as a length-based weight estimation tool in India is two-fold. Emergency medicine is an emerging field in India and an age-based weight estimation in the pediatic population during emergency intervention is more prone to dosing errors. In its current format, however, the Broselow tape cannot be utilized with optimal accuracy. The Indian children are underweight for their age and height.

The Broselow tape is deemed a practical tool that facilitates a fast weight estimation in emergency settings and may help to circumvent dosing errors. Recent studies provide ample evidence on dosing errors in pediatric emergency settings with concomitant large percentage of adverse events (AE) [1–4]. Manual dose calculation using dosing equations during pediatric treatment also proved to be a high-error activity [5–8]. The Broselow tape was developed for use in the USA to overcome such impracticalities.

In India, the age-derived weight methods using the Nelson formula or APLS in pediatrics is often used [9, 10]. However, recent studies in India indicate that such method may be erroneous.

A comparison of age-derived methods with length-based estimation of weights has demonstrated that the Broselow tape can predict dosages of resuscitation drugs with better accuracy [11]. Evidence shows that the APLS formula tend to underestimate the actual weight and the margin of error increases with age [12].

A comparative study on the Luscombe and Owens new formula and the most recent new APLS formulae has not been conducted in India. The new APLS guidelines include three new formulae that stratify the pediatric population using the original APLS for ages 1–5 years and Luscombe and Owens formulae for ages 6–12 years [13]. The Broselow tape is recommended by the Indian Academy of Pediatrics [14]. A recent study has provided us with compelling evidence that methods based on the length of the child are more accurate than age-based formulae [15].

Pediatric medication dosing has been recognized as a high-error activity with the potential to cause serious harm, and it has been found that the Broselow tape and color-coded materials would result in a decrease in deviation from recommended medication doses and equipment sizes and an increase in physician comfort level [16].

The Broselow tape correlates measured heights ranging between 46–146.5 cm to predicted weight ranges between 3–36 kg, arranged in color zones. Its predicted weight ranges are derived from pediatric anthropometric data collected by the National Center for Health Statistics (NCHS) between 1963–1975. The color zones are designed to predict the 50th percentile weight for height, which is an estimate of ideal body mass [17]. The tape is recommended by the Advanced Trauma Life Support (ATLS) [18] and the Pediatric Advanced Life Support in the USA and Europe [19]. The use of the Broselow tape has been the subject of several studies to validate its use [20–23].

Investigation on the Broselow tape in the USA and other countries have identified either an underestimation or overestimation of body weight in pediatric emergency settings. In the USA, several studies have demonstrated an *underresuscitation* phenomenon. The American pediatric population is overweight for their height [24]. For this reason, the Broselow tape in the USA generates weight estimates that are found to be within 15 % of error for 79 % of children and showing a high degree of accuracy for children from 3.5 to 10 kg and from 10 to 25 kg [25].

On the contrary, the tape *overestimates* weights for the Indian pediatric population and would pose the risk for *overresuscitation* in an emergency setting. A previous prospective study determined that the Broselow tape overestimates weights in the Indian pediatric population by >10 % [26].

In our study, we validated the overestimation of predicted weights by the Broselow tape among the Indian children and remodeled it as a new Indian pediatric weight estimation tool (IPWET) that has new weight ranges and enhanced sensitivity.

## Methods

In this prospective observational study, 1185 children of both sexes were recruited in two phases: phase 1 (*n* = 769) and phase 2 (*n* = 416) at the Sri Ramachandra University Hospital, Department of Pediatrics, Chennai, India. All participants met the inclusion and exclusion criteria. This study was carried out during July–August 2009, after obtaining an informed consent from the parents or accompanying guardians. This study was approved by the Institutional Ethics Committee of University hospital. The aim of the study was to remodel the Broselow tape for the Indian children. Indian children are underweight for their age and height compared to the 50 percentile data represented on each color zone of the Broselow tape.

### Study setting and population

This study was conducted at Outpatient Pediatrics Department at a Sri Ramachandra University hospital in India. This tertiary hospital provides care for nearly a thousand pediatric patients per day with diverse socioeconomic backgrounds.

#### Inclusion criteria

The Pediatric population, aged 1 month to 12 years including both genders.

#### Exclusion criteria

Children having length <46 cm or height >146 cm, weighing > 36 kg, severely malnourished, or dehydrated children, and children requiring emergency care.

### Study protocol

The study protocol was reviewed and approved by the University Ethics Committee. Subjects’ name, age, date of birth, and reason for the visit to the clinic was recorded before the actual measurements of height and weight were taken. The weight was measured by using a digitalized weighing scale to the nearest 0.1 kg. Prior to measurement, the footwear and heavy clothing were removed. Consequently, the subject was invited to assume a supine position on a bench parallel to the Broselow tape. We used a *Broselow Pediatric Emergency Tape*, 2007, Edition B, Armstrong, Medical Industries, IL, USA.

Two pieces of cardboard were used to hold against the crown and heel of the subject. The red arrow on the tape was held by the crown perpendicular to the cardboard, and the predicted weight was read from the corresponding color zone and particular weight on the Broselow tape. In a similar manner, a standard measuring tape was used to measure the height of the subject and match the height to corresponding color zone. In case of children under age 1 year or those who were still too young to stand, the measurement of weight was obtained by placing the child on the infant scale. The height was measured by placing the infant on the bed in a straight supine position and holding two cardboard pieces against the crown and heel of the infant.

Prior to commencement of data collection, the scales were calibrated according to manufacturer guidelines. Ten percent of the data was measured by two investigators in blinded fashion to determine the interrater reliability.

### Outcome and measures

The outcome measures for the performance of the Broselow tape included a mean percentage difference as an estimation of bias, standard deviation (SD) as a measure of precision, and percentage agreement on color-coded zones and prediction of weights based on the measured height (within 10 % of the measured weight) as a measure of tape accuracy.

### Data analysis

Linear regression, ANOVA, *t* statistics, *p* value calculation, kappa value, chi-square, specificity, and sensitivity diagnostic tests were applied to analyze the data. SPSS 15.0 (SPSS Inc., Chicago, IL, USA) was used to analyze the data.

## Results

Of the selected 769 children, 52.9 % (407) are boys and 47.1 % (362) are girls. The difference between the gender representation is not significant (*p* = 0.113). The distribution of boys and girls is equivalent in the selected subjects (Table 1). Our sample data is statistically sufficient for analysis with respect to height and weight. The correlation coefficients for the anthropometric measures in our study included the following: (a) measured weight vs. measured height (0.954), (b) measured weight vs. the Broselow tape weight (0.970), (c) measured height vs. the Broselow tape (0.978), and (d) weight measured weight vs. age (0.925). These measures indicate a high degree of positive association between actual weight and actual height with observable gender relationships between variables and show similar coefficients with a *p* value <0.0005.

**Table 1:** Descriptive statistics and selected characteristics of the subjects (*n* = 769)

Characteristic	Mean	SD
Age (months)	52.66	43.21
Actual weight (kg)	14.29	7.54
Actual height (cm)	96.64	26.06
Broselow weight (kg)	15.51	8.43
Estimated weight for height (from the data)	14.29	7.19
Mean weight (kg) (Broselow and Actual)	14.89	7.92
Difference in weight (kg) (Broselow − Actual)	1.23	2.15
Percent difference in weight (Actual − Broselow)	−8.49	14.59
Absolute Percent difference in weight (Actual − Broselow)	12.88	10.92

**Fig. 1:**
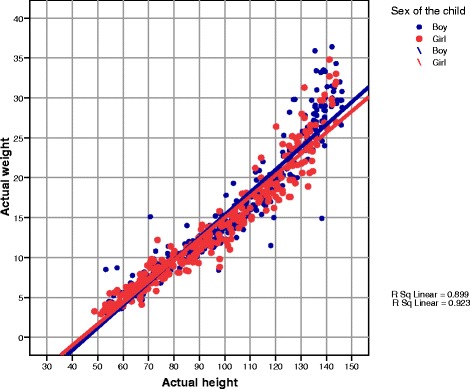
Scatter plot of measured heights and measured weights (kg)

The relationship between actual height and weight for boys and girls, together with the regression line, is illustrated in Fig. 1. The coefficient of determination for boys (blue dots) is 0.899 and for girls (red dots) is 0.923, which shows the greater applicability of the values. The linear regression shows concordance between measured weights and predicted weights on the Broselow tape.

**Fig. 2:**
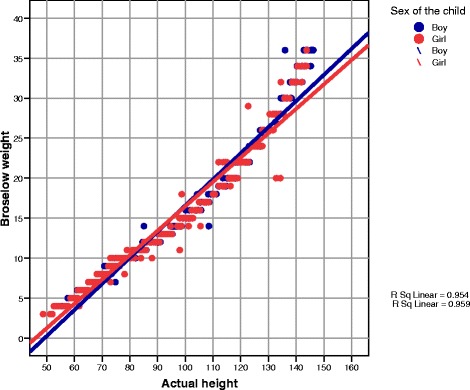
Scatter plot of measured heights and Broselow predicted weights (kg)

The association between actual heights and the Broselow predicted weights has a coefficient of determination of 0.954 for boys and 0.9 for girls (Fig. 2), and *R*^2^ shows 95.4 and 90 % variability. The heights in the data sample are not congruent with the purported predicted weights by the Broselow tape. The difference between the actual weights and the predicted weights from the Broselow tape demonstrates <100 % agreement. The Broselow tape overestimates predicted weights with a mean percentage difference of 5–15 % depending on the color zone (Table 3). The accuracy by color zone was between 33–86.6 %, with higher weight color zones showing lower accuracy. There is an 8 to 14 % mismatch of measured heights to color zones on the Broselow tape (Table 2).

**Table 2:** Sensitivity, specificity, kappa, and chi-square level of significance for different color zones on Broselow tape with reference to the actual weight of the children (*n* = 769) prior to correction

Color code	Sensitivity (%)	Specificity (%)	Kappa	Chi-square *P* value
Gray	69.1	98.7	0.73	0.0005*
Pink	76.3	96.4	0.72	0.0005*
Red	51.5	94.0	0.47	0.0005*
Purple	33.0	94.2	0.30	0.0005*
Yellow	68.6	93.1	0.60	0.0005*
White	54.8	94.6	0.52	0.0005*
Blue	57.1	94.1	0.51	0.0005*
Orange	48.1	93.6	0.41	0.0005*
Green	86.8	94.5	0.57	0.0005*

*p < 0.05 (*p* value is highly significant)

The overall difference between estimated Broselow predicted weights and actual weights in our data sample for each color zone falls within 8.49 % (Broselow weight − actual weight) × 100/actual weight). We have truncated the value to 8 % and used it as a correction factor to enhance sensitivity and the degree of agreement between measured heights and predicted weights on the tape. The correction factor reduces the Broselow predicted weights for each color zones by 8 % (Table 4). Sensitivity and kappa values demonstrate the agreement between measured weights and predicted weights. The kappa value quantifies the level of agreement between the actual weights and the Broselow predicted weights for each color-coded zone. The kappa values for our sample data are all positive and congruent with the sensitivity values. The values are >0.7, except for red, purple, and orange zones (Table 2), and statistically significant. The kappa value is not 1 because the sensitivity is not 100 %. In our scenario, *sensitivity* is the tape’s capacity to correctly identify and match a given height to the correct color zone on the tape. Specificity, on the other hand, is the tape’s capacity to differentiate color zones and should not assign height to the wrong color zone. Sensitivity and specificity are measures of the tape’s accuracy (Table 2).

In our data sample, the Broselow tape misclassified the heights to inaccurate predicated weights with a mean percentage difference of 5–15 % depending on the color zone. Accuracy by color-coded zone was between 33–86.6 %, with higher weight color zones showing lower accuracy. With an 8 % correction factor, the mean percentage difference was reduced by 4–7 % and reflects an improved accuracy between 51–97.8 % (Table 3, Table 4).

**Table 3:** Mean percentage difference for color zones (*n* = 769) measured weights and Broselow predicted weights before and after correction

Color	*N*	Mean before correction (SD)	Mean after 8 % correction (SD)
Gray	55	16.37 (20.49)	7.05 (18.84)
Pink	93	4.30 (12.98)	−4.04 (11.94)
Red	97	7.37 (17.11)	−1.2 (15.75)
Purple	91	4.39 (12.55)	−3.95 (11.55)
Yellow	121	8.81 (12.29)	0.11 (11.31)
White	104	9.86 (17.85)	1.07 (16.42)
Blue	91	11.46 (10.17)	2.54 (9.36)
Orange	79	10.78 (10.86)	1.91 (9.99)
Green	38	3.49 (11.21)	−4.79 (10.31)
Total	769	8.49 (14.59)

**Table 4:** Average measured weights, Broselow predicted weights, and 8 % reduced Broselow weights

Color zone	Measured weight (SD)	Broselow weight (SD)	8 % less Broselow weight (SD)	*P* value	*P* value
				(Column 2 vs. Column 3)	(Column 2 vs.Column 4)
1	2	3	4	5	6
Gray (78)	5.02 (1.3)	4.94 (0.97)	4.54 (0.89)	0.50	0.0005
Pink (136)	7.71 (1.81)	7.74 (0.85)	7.12 (0.78)	0.80	0.0005
Red (55)	8.79 (1.04)	9.56 (0.69)	8.79 (0.63)	0.0005	0.98
Purple (113)	11.17 (1.36)	12.07 (0.91)	11.1 (0.84)	0.0005	0.44
Yellow(107)	13.8 (1.89)	14.95 (1.3)	13.76 (1.2)	0.0005	0.73
White (105)	17.69 (2.17)	19.58 (1.88)	18.01 (1.73)	0.0005	0.063
Blue (86)	22.7 (3.11)	24.72 (2.12)	22.74 (1.95)	0.0005	0.89
Orange (39)	26.8 (3.44)	29.33 (2.26)	26.98 (2.08)	0.0005	0.69
Green (50)	29.32 (3.45)	33.52 (1.83)	30.84 (1.68)	0.0005	0.003

**Fig. 3:**
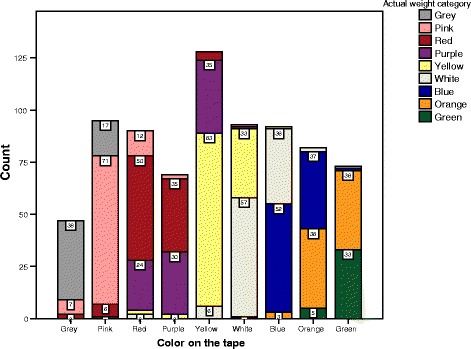
Color-coded zones and number of children matching in actual weights in color zones of Broselow predicted weights before correction factor of 8 %

We have quantified the overestimation of predicted weights by the Broselow tape in terms of *overlapping* of one color zone (Fig. 3). It is indicative of an inadequate concordance between measured height and predicted weights. The majority of overlapping occurs in one higher color zone.

The 8 % correction factor also enhanced the sensitivity and produced a uniform high level of specificity. The sensitivity rate is the highest on either end of the tape (gray and green colors), while the middle zone shows greater sensitivity (purple and orange colors), and the red, white, and blue colors have moderate sensitivity values.

The difference between measured weight and the Broselow predicted weight has a significant *p* value. The correction factor reversed the significance of the *p* value (Table 4, Columns 5 and 6). The gray and pink color zones measured weights are in close agreement with the Broselow predicted weights. However, as we transition to other color zones, the mean Broselow predicted weight is more than the measured weights, and the difference is statistically significant. Furthermore, the disagreement is in favor of measured weight. In other words, measured weights are greater than the predicted weights on the corrected Broselow values.

Applying ANOVA, we determined 95 % confidence interval for the measured heights for each color zone on the Broselow tape. Each color zone on the Broselow tape has a height range that does not match the measured heights of the selected sample children. In order to correct or remove the overlapping of the height, we chose the 95 % CI of measured heights in the Broselow color zones, and we have taken these new height ranges as the *reference height* ranges. As a result of the exercise, we reduced the overlapping of color zones (Table 4). We used the reference height ranges in a univariate generalized linear model to determine their corresponding new *weight ranges* (Table 4 and Table 7).

### Sample size

To determine the number of new sample data for testing our new tape, we have taken the overlapping phenomenon, depicted in Fig. 3, by excluding 20 % of data on both sides of each color zone (preceding and succeeding areas), we isolated the middle zone as such that would contain non-overlapping values for each color zone. The gray zone is an exception, as there is no preceding color zone. In the process, we have determined that we need a minimum of 40 samples per color zone with a significance level of 5 % and a power of 80 %. This value translated into 360 samples for nine color zones. We used data from 769 children to drive the new height and weight ranges for IPWET.

### Validation of the new height and weight ranges

We recruited an additional 416 children of both genders at the same study site to validate our findings. Our analysis confirmed that the adjusted Broselow tape (IPWET) indeed enhances the degree of agreement and kappa value with a stronger correlation between measured heights and estimated weights (Table 5 and Table 6).

**Table 5:** Sensitivity values for height and weight ranges of the data samples

Color	Color	Sample-1	Sample-1	Sample-2	Sample-2	Overall sample	Overall sensitivity
(height range)	(weight range)	(*n* = 769)	Sensitivity	(*n* = 416)	Sensitivity	(769 + 416 = 1185)	
Gray(<64.99)	Gray (<6.49)	78	68.4	46	100	124	78.3
Pink(65–73.99)	Pink (6.5–8.49)	136	60.2	48	94.3	184	69.2
Red(74–80.99)	Red (8.5–10.49)	55	25.7	28	40.7	83	31.0
Purple(81–94.99)	Purple (10.5–12.49)	113	63.2	67	72.5	180	66.4
Yellow(95–106.99)	Yellow (12.5–15.49)	107	61.2	44	57.4	151	59.9
White(107–120.99)	White (15.5–19.49)	105	68.1	61	90.7	166	75.2
Blue(121–132.99)	Blue (19.5–24.49)	86	60.5	45	65.6	131	62.6
Orange(133–137.99)	Orange (24.4–28.49)	39	36.0	31	48.5	70	41.0
Green(138–150)	Green (28.5–36.00)	50	69.4	46	82.5	96	75.3
Total		769		416		1185

**Table 6:** Kappa values for individual color zones on the remodeled tape

Color	Color	Sample-1	Kappa for	Sample-2	Kappa for	Overall sample	Overall kappa
(height range)	(weight range)	(*n* = 769)	769	(*n* = 416)	416	(769 + 416 = 1185)	
Gray (<64.99)	Gray (<6.49)	78	0.73	46	0.988	124	0.819
Pink (65–73.99)	Pink (6.5–8.49)	136	0.42	48	0.733	184	0.518
Red (74–80.99)	Red (8.5–10.49)	55	0.27	28	0.492	83	0.343
Purple (81–94.99)	Purple (10.5–12.49)	113	0.51	67	0.567	180	0.531
Yellow (95–106.99)	Yellow (12.5–15.49)	107	0.52	44	0.584	151	0.542
White (107–120.99)	White (15.5–19.49)	105	0.59	61	0.716	166	0.633
Blue (121–132.99)	Blue (19.5–24.49)	86	0.56	45	0.72	131	0.617
Orange (133–137.99)	Orange (24.4–28.49)	39	0.37	31	0.458	70	0.406
Green (138–150)	Green (28.5–36.00)	50	0.67	46	0.741	96	0.701
Total		769		416		1185	

### Limitations

Although our data sample is diverse and representative of all geographic regions and socioeconomic backgrounds in the Indian population, it is collected at one tertiary clinic. There is no comparable NCHS data pool available in India that would allow a precise estimate of height and weight for each color zone. Therefore, our correction factor may change at the aggregate or individual color zone level when we repeat the study on a very large and diverse pediatric population. In this study, we have considered only gender difference, and other categories such as social class, income, parent education level, differences in ethnicity, and regional areas are not considered in the analysis. We could not with certainty verify the age of the children in our data sample and relied on the information obtained from the parents or guardian. The future study should include Bland Altman plot and a measurement error model to provide a more informative comparison by determining the relative biases (i.e., systematic error, including differences in measurement scale, or scale bias) and imprecision (i.e., random error, adjusted for scale bias).

## Discussion

Our discussion is limited to factors that may explain why Indian children are underweight for their height and support our effort to remodel the Broselow tape. Our results confirm that the Broselow tape overestimates weights with a 5–15 % mean percentage difference in the Indian children and misclassifies them into a wrong color zone on the Broselow tape, usually to a higher color zone. This finding implies *overresuscitation* with inaccurate dosing of emergency medications.

As the weights (kg) in our data sample decreases with increase in age, the level of sensitivity declines as well. This observation is indicative of a trend that with increased age, the Indian children become more underweight for their heights (Table 2).

There are several possible reasons for this trend. Numerous surveys and studies have focused on the level of nourishment, exclusive breast-feeding (EBF), socioeconomics, and low birth weight. In our current analysis, we devoted our attention to these contributory factors to highlight current data and their relevance to lag in ideal height-for-weight and weight-for-age in the Indian pediatric population.

In a recent survey in southern India, where our study was conducted, one study has determined that the prevalence of undernutrition (≤80 percentage of standard weight for age) was 66.5 %. Such prevalence tends to increase with age and there is a stark difference between male (76.9 %) and female (56.3 %) genders. The prevalence of grade 1 malnourishment was 46.2 %. The prevalence decreased as the socioeconomic status improved and the duration of EBF had an influence on the nutritional status of the growing child [27].

The breast-feeding policy, instituted in 1992, and the Global Strategy for “Infant and Young Child Feeding” (IYCF) initiated in 2002, have not helped to reduce the level of Moderate Acute Malnutrition (MAM) and Severe Acute Malnutrition (SAM) including Edematous SAM (E-SAM) among children in India under 5 years of age. Recently, the National Family Health Survey (NFHS-3) in India revealed staggering data assessing child growth that showed 43 % of children were underweight, 48 % were stunted, 20 % were wasted, and 7.9 % had severe wasting [28]. The NFHS-3 survey during 2005–2006 concluded that the rate of EBF at 6 months of age in the Indian infants was only 46.4 % [29].

WHO and UNICEF recommend the use of secondary source of human milk as the first alternative for mothers unable to breast-feed. In 2013, India took initiatives to draft comprehensive guidelines to establish human milk banks (HMB). Today, there are only 14 HMB throughout India to provide human milk for mothers who are unable to adequately breast-feed their infants [30].

Another unique challenge to the growth of children is the high rate of very low birth weight (VLBW) in India (20 %) that causes a significant mortality and morbidities and most probably affects the normal growth [31]. Pregnant women in resource-poor areas are at risk of multiple micronutrient deficiencies, and indicators of low Vitamin B_12_ status have been associated with adverse pregnancy outcomes, including anemia, low birth weight, and intrauterine growth retardation [32, 33]. Results show that mother nutritional status is the most important determinant of newborn child birth weight in India. Safe drinking water, use of antenatal care, and iron deficient anemia were also significant contributors to low birth weight. In addition, the mother BMI impact is more pervasive across India than the impact of other factors on birth weight [34].

Furthermore, there is a correlation between nutritional knowledge, maternal education status, and birth weight in poor communities in the Indian urban slum areas. In one pilot study, nutritional grading was done through a scoring system to demonstrate the relationship among the aforementioned factors. The study showed 47 mothers (24.1 %) out of 195 mothers having normal nutritional grade while 37 mothers (19 %) having severe nutritional grade. There were 80 mothers having “no knowledge of maternal nutrition.” Adequate knowledge was found in 31 cases. Mothers (*n* = 80) with “no knowledge of maternal nutrition” delivered babies of average weight 2.3 kg, whereas “adequate knowledge” mothers gave birth to babies of average weight 2.9 kg [35].

Several studies have cited the benefits of early breast-feeding and its link with a reduced risk of obesity compared with formula feeding [36]. There is a correlation between the percent of maternal ideal weight and lipid concentration in maternal milk during 6–12-months postpartum growth period that is linked to subsequent leaner body mass in the growing child [37]. However, analysis of data linking the impact of breast-feeding and lower body mass in the Indian pediatric population does not exist. In general, breastfed children in the developing world can achieve similar growth as those in the developed world when similar socioeconomic and nutritional criteria are met [38].

Currently, three quarters of the Indian pediatric population are underweight. During 1998–1999, a study found over 73 % of Indian children <3 years of age to be underweight for their height [39, 40]. Such findings are related to socioeconomic status among Indian children, and children from affluent status show similar growth patterns prevalent in the developed countries [41]. In the past five decades, community medicine in India has made tremendous strides in the field of preventive medicine. We are convinced that the lag behind the normal growth curve in the Indian pediatric population will change as new and more rigorous growth promoting strategies are being implemented. In the future, similar to the readjustment of the Broselow tape for the children in the USA due to a higher prevalence of obesity, our remodeled tape will be readjusted for the Indian children as their height-for-age and weight-for-age change due to changes in their nutrition status.

## Conclusions

Our remodeled tape (IPWET) has a higher accuracy between 51–97.8 % compared to 33–86.6 % accuracy rate of the Broselow tape that decreases with higher weight color zones when used for the Indian pediatric population. The new tape has a weight range of 4–36 kg and a height range of 50–150 cm vs. weight range of 3–36 kg and height range of 46–146.5 cm on the Broselow tape (Table 7).

**Table 7:** Broselow predicted weights and heights vs. new estimated weights and corresponding heights for Indian pediatric population

Color zone	Broselow	Broselow	New	Estimated
	height range (cm)	weight range (kg)	height range (cm)	weight range (kg)
Gray	46–59.4	3–5	50–64.99	4–6
Pink	59.5–66.5	6–7	65–73.99	7–8
Red	66.5–74	8–9	74–80.99	9–10
Purple	74–84.5	10–11	81–94.99	11–12
Yellow	84.5–97.5	12–14	95–106.99	13–15
White	97.5–110	15–18	107–120.99	16–19
Blue	110–120.75	19–23	121–132.99	20–24
Orange	120.75–133.4	24–29	133–137.99	25–28
Green	133.4–146.5	30–36	138–150.00	29–36
